# Abdominal-Based Microsurgical Breast Reconstruction: How to Inset the Flap to Maximize the Aesthetic Result—A Systematic Review

**DOI:** 10.3390/jcm12196135

**Published:** 2023-09-22

**Authors:** Gianluca Sapino, Sherilyn K. Tay, Michele Maruccia, Lloyd Nanhekhan, William Watfa, Gian Piero Mantovani, David Guillier, Pasquale Tedeschi, Russell Bramhall, Pietro Giovanni Di Summa

**Affiliations:** 1 Department of Plastic and Hand Surgery, Centre Hospitalier Universitaire Vaudois (CHUV), 1011 Lausanne, Switzerlandl.nanhenkhan@gmail.com (L.N.); 2 Canniesburn Plastic Surgery Department, Glasgow Royal Infirmary, Glasgow G4 0SF, UK; sherilyn.tay@nhs.net (S.K.T.); russell.bramhall@ggc.scot.nhs.uk (R.B.); 3 Department of Plastic and Reconstructive Surgery, University Hospital of Bari, 70124 Bari, Italy; marucciam@gmail.com (M.M.);; 4 Department of Plastic and Reconstructive Surgery, Saint George University Hospital, Beirut 1100, Lebanon; william.watfa@gmail.com; 5 Department of Plastic and Reconstructive Surgery, University Hospital of Modena, 41121 Modena, Italy; gpieromanto@gmail.com; 6 Department of Plastic Reconstructive and Hand Surgery, Department of Oral and Maxillofacial Surgery—University Hospital, 21000 Dijon, France; docteurguillierdavid@gmail.com

**Keywords:** breast reconstruction, diep flap, aesthetic reconstruction

## Abstract

Nowadays, the ultimate goal of microsurgical breast reconstruction is not merely the effective transfer of vascularized tissue but the achievement of a natural, symmetric appearance. The aim of this present study was to systematically summarize the published evidence on abdominal-based free flap inset for breast reconstruction in order to provide principles and classification that could guide the surgeon in choosing the most appropriate inset technique based on patient and flap characteristics. A comprehensive review was performed following the Preferred Reporting Items for Systematic Reviews and Meta-Analyses (PRISMA) guidelines, looking for articles on the insetting technique for free flap breast reconstruction. After screening 306 publications, 24 papers (published from 1994 to 2020) were included in the study. We identified four main breast anatomical features on which the papers reviewed focused when describing their insetting technique: breast width, breast ptosis, breast projection, and upper pole fullness. Patient body type, type of mastectomy, and reported complications are also discussed. Flap shaping and inset during breast reconstruction are fundamental steps in any reconstructive procedure. Despite the low evidence in the current literature, this systematic review provides a framework to guide the surgeon’s decision-making and optimize the aesthetic outcomes of abdominal-based free flap breast reconstruction.

## 1. Introduction

Breast reconstruction is considered worldwide to be a fundamental part of breast cancer treatment and is associated with improved psychosocial wellbeing following breast cancer survival [1].

Medical and surgical advancements over the years have led to continuously improving quality of life outcomes. Autologous microsurgical breast reconstruction can provide long-lasting results with a natural shape that ages physiologically over time. The avoidance of breast implants and their related complications (e.g., implant rupture, capsular contraction, and more recently, BIA-ALCL) has made autologous breast reconstruction not only the most natural way to reconstruct the breast but often also the most economic long-term reconstructive option due to the avoidance of implant-related maintenance costs [2,3,4].

The popularization of free tissue transfer and the continuous development of microsurgical techniques have made breast microsurgical reconstruction an extremely safe procedure with minimal failure rates when an experienced microsurgical team is present [5]. Indeed, when assessing breast reconstruction outcomes, the final aesthetic result and near-perfect simulation of the contralateral native breast have become the new surgical goals.

The abdomen remains the preferred donor area for autologous breast reconstruction. Since the description of the transverse rectus abdominis myocutaneous (TRAM) flap, the refinement of microsurgical techniques has evolved to reduce donor site morbidity through the development of the deep inferior epigastric perforator flap (DIEP) and the superficial inferior epigastric artery flap (SIEA), as well as multi-pedicled and stacked flaps [6].

When shifting attention from tissue survival and optimal perfusion to flap insetting, multiple strategies have been proposed in the literature [7,8]. However, publications are often limited to small series without a global approach describing how to achieve the ideal final shape and appearance of the new breast. The lack of this more systematic approach when describing insetting procedures is due to the multitude of reconstructive parameters to consider (such as contralateral breast shape, flap donor site features, and type of mastectomy), making one single insetting method insufficient to fit all clinical situations. Indeed, despite the various techniques for abdominal flap inset that have been published for breast reconstruction, a systematic review is still lacking in the literature [9].

The aim of this present study is to systematically summarize the published evidence on abdominal-based free flap inset for breast reconstruction in order to provide principles and classification that could guide the surgeon in choosing the most appropriate inset technique based on patient and flap characteristics.

## 2. Materials and Methods

A comprehensive review was performed following the Preferred Reporting Items for Systematic Reviews and Meta-Analyses (PRISMA) guidelines, to find articles on the insetting technique for free flap breast reconstruction. The search was conducted between November and December 2020, using the PubMed database. Keywords used were as follows: breast AND free flap AND (insetting OR modeling OR shaping). Both medical subject heading (MeSH) terms and free-text terms were used to construct the search algorithm. There was no restriction on the time of publication, and only English-written articles were retained.

The inclusion criteria for this review consisted of the following: (1) either a case study, a case report, a case series, a clinical trial, an open-label prospective study, or a retrospective study; (2) papers focused on breast reconstruction with free flaps in which the inset technique was clearly defined. The exclusion criteria were as follows: (1) literature reviews and letters; (2) publications in which it was impossible to determine the specific technique of free flap inset; (3) articles with unclear presentations of outcomes and complications; (4) studies describing pedicled flap reconstruction. Only abdominal-based free flap breast reconstructions were included due to the low number and significance of back/lower limb-based breast reconstructions relevant to the scope of the review.

All publications were screened manually. Three investigators (G.S., W.W., and D.G.) independently reviewed and extracted data from the papers according to the predetermined criteria. In addition to the above-mentioned database searches, reference lists of included studies were manually cross-referenced by the first author (G.S.) to retrieve additional articles eligible for inclusion.

**Figure 1:**
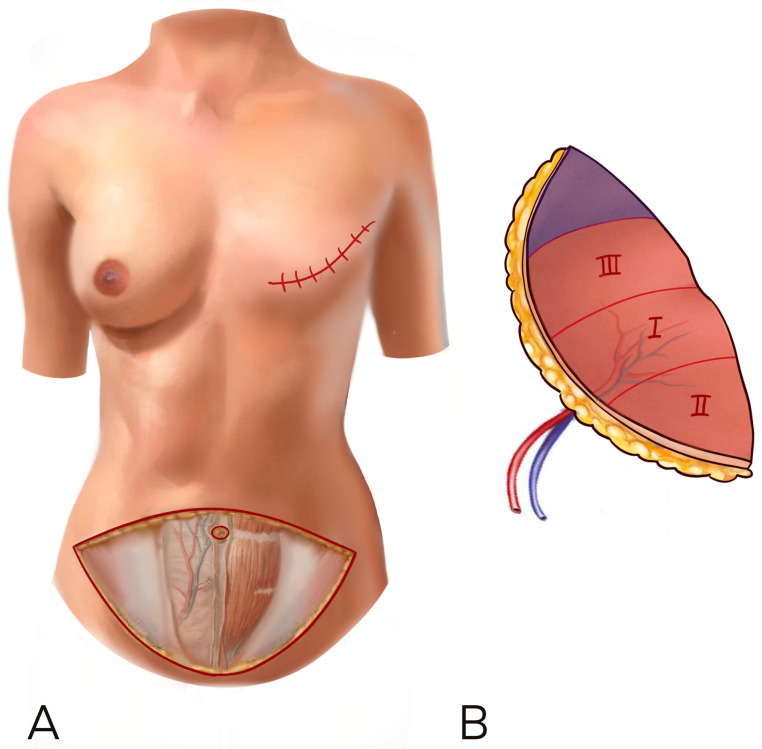
(**A**,**B**) The Holm perfusion zones of the DIEP to display our result: zone I is where the perforator enters, zone II is ipsilateral to the perforator chosen, while zone III is across the midline.

According to previous literature and for uniformity of data collection, we decided to use the Holm perfusion zones of the DIEP to display our result: zone II is ipsilateral to the perforator chosen, while zone III is across the midline [10] (Figure 1).

The following information was documented and tabulated for each article: author name(s), year of publication, number of patients, age of patients, type of flap, insetting technique, microsurgery data, outcomes, and complications. Key recurring themes were identified across the included papers.

## 3. Results

**Figure 2:**
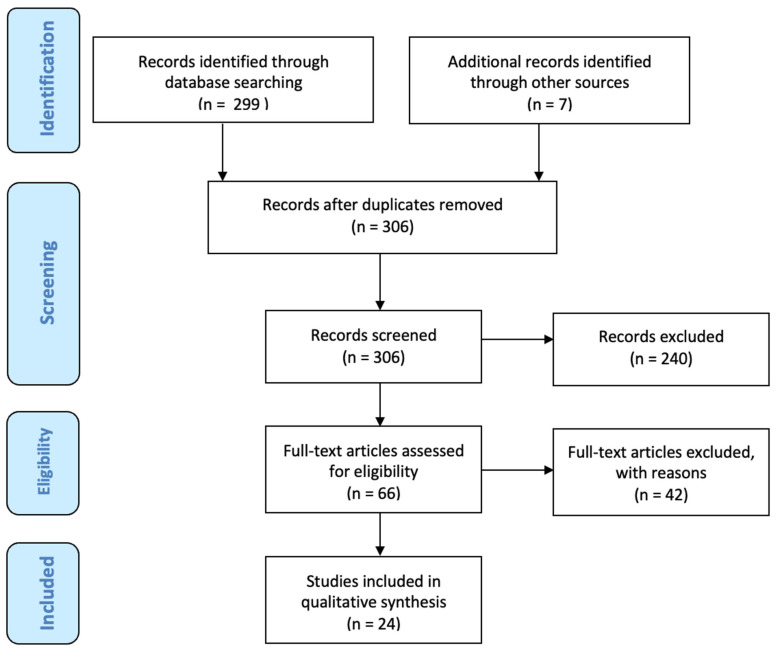
The PRISMA Flowchart.

After screening 306 publications (including 7 papers describing lower limb/back-based flap reconstruction, which were excluded as stated in the Section 2), 66 articles were selected for full-text review. Among these, 17 papers matched the inclusion criteria and were analyzed comprehensively. In total, 7 articles were further included from the references, bringing the total number of papers included to 24, published from 1994 to 2020 (Figure 2). There were 16 retrospective studies, 2 prospective studies, and 6 case reports. Overall, 1213 patients and 1450 abdominal free flaps were represented in this review. Among the abdominal flaps, there were 1252 DIEP flaps, 182 TRAM flaps, and 15 SIEA flaps. In 239 patients (20%), a stacked-bipedicled flap reconstruction was performed. The timing of reconstruction was delayed in 479 patients (40%) and immediate in 742 patients (60%). See Table 1 for a summary of the studies included in the study.

### 3.1. Delayed Reconstructions

The type of mastectomy (skin sparing vs. nipple sparing) and the timing of reconstruction (delayed vs. immediate) influenced the insetting technique.

In delayed reconstructions, two critical features could be highlighted after literature critical analysis: the role of the inframammary fold (IMF) and the amount of available skin.

The position of the new IMF is of paramount importance to defining the lower border of the breast footprint [32]. The original IMF may be difficult to identify, as the oncologic surgeon may have violated it during the mastectomy. Authors agreed on placing the new fold slightly higher than the contralateral one (around 2 to 3 cm) in anticipation of the abdominal closure, which is thought by some authors to pull the IMF caudally and otherwise lower it. When in doubt, the new IMF should be better placed a little higher than too low, as the correction of a higher IMF is much easier [12].

According to Blondeel et al., the skin between the mastectomy scar and the newly defined IMF should be removed while preserving as much of the subcutaneous layer as possible, as this will improve the projection of the breast lower pole [33,34]. It is also often considered advantageous to replace poor-quality chest skin, which can result in a tight skin envelope compressing the buried flap with an unnatural appearance. Positioning more of the flap under the upper mastectomy skin flap reduces the final amount of ptosis of the reconstructed breast but is often performed to improve upper pole fullness. Some authors underlined the fact that eliminating the lower mastectomy flap (from the mastectomy scar to the new IMF) will reduce the appearance of the skin patch as the whole breast is reconstructed as a single aesthetic unit [24]. Other authors suggest that the lower mastectomy flap should be preserved, raised, and split in the breast meridian to improve breast ptosis while also maintaining good projection. This option was particularly useful in patients declining a contralateral mastopexy [34].

### 3.2. Breast Key Features and Inset Rules

We identified four main breast anatomical features [35] on which the papers reviewed focused when describing their insetting technique:

- breast width
- breast ptosis
- breast projection
- upper pole fullness

#### 3.2.1. Breast Width

Several authors highlighted that the lower abdomen harvested for breast reconstruction has an elliptical shape with relatively predictable geometric characteristics [14,24]. Flap thickness is maximal medially and cranially near the umbilicus. Most flaps are thinner laterally and caudally/in the pubic area. The skin paddles in zones I and III are wider (distance from the upper and lower flap borders) in the middle of this ellipse than zones II and IV at the tips of the ellipse [23].

A horizontal inset to enhance width is preferred when a large neo-breast needs to be reconstructed. This can be particularly indicated in patients with a higher BMI where the base of the contralateral breast exceeds 20 cm, in older patients where the contralateral breast naturally falls more laterally, in patients with particularly long mastectomy scars (in secondary/delayed reconstructions), or finally where lateral tissues have been particularly damaged by radiotherapy and benefit from being replaced by well-vascularized flap tissue [9].

The authors did not agree on a preference for flap harvest laterality. The preference was instead to use the best perforator, regardless of laterality.

**Figure 3:**
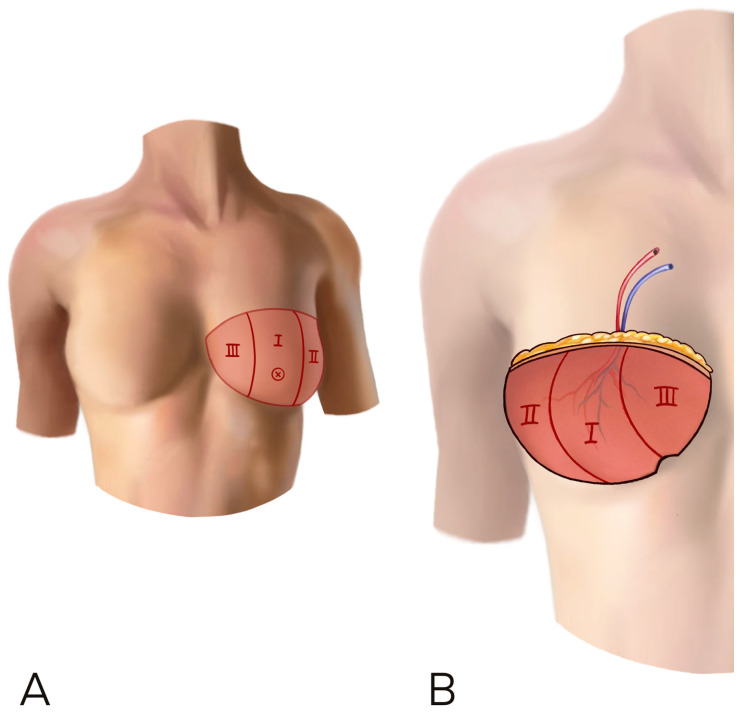
(**A**,**B**) A horizontal inset is shown. The flap was raised on a perforator contralateral to the recipient site and the flap has been rotated 180° in order to have the umbilical part of the flap on the inferior bord of the breast. This inset can add more thickness at the lower and medial pole of the neo-breast.

When choosing a medial perforator ipsilateral to the reconstruction side, the flap can be transferred to the chest area without rotation, keeping the better perfused part of the flap in the medial part of the breast. When the perforator is contralateral to the breast to be reconstructed, the flap is better rotated to leave the thinner, lateral flap tip in the axilla, avoid excessive lateral bulk, and again maintain medial fullness (Figure 3).

The majority of the authors insisted on internal flap fixation with absorbable sutures; three key sutures can be placed afterwards in the supero-lateral, midclavicular, and inferomedial parts of the flap. The supero-lateral suture should be placed 2-3 cm medial to the lateral border of the pectoralis major; shifting this suture more laterally would increase the lateral bulk and breast base width. It is worth noting that the flap will tend to shift laterally over time [13,22,23].

#### 3.2.2. Breast Ptosis

Ptosis represents one of the anatomical characteristics of a natural breast and is often the key feature differentiating an autologous reconstruction from an implant-based reconstruction.

**Figure 4:**
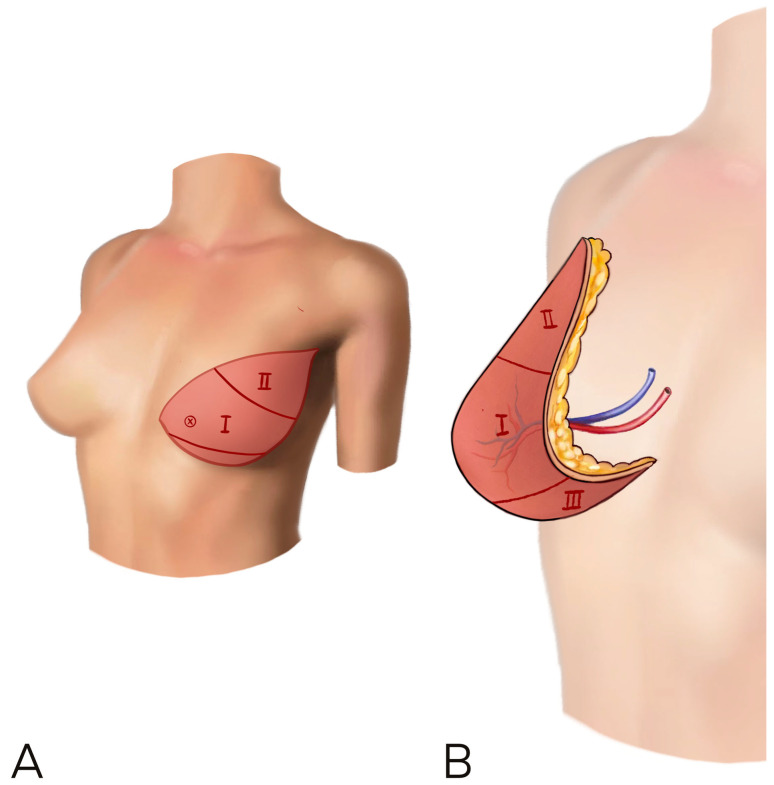
(**A**,**B**) A vertical rather than horizontal skin paddle inset should be considered to increase the skin available vertically and improve ptosis. The flap is rotated to let the umbilical and thicker part in the medial side of the breast, in order to improve cleavage and reduce lateral bulk.

When more breast ptosis is required, a vertical rather than horizontal skin paddle inset should be considered to increase the skin available vertically and thus improve ptosis (Figure 4) [9].

Jeong et al. described using the thicker cranial/medial part of the flap in the lower pole and securing the thinner flap tip (Zone II) superolaterally to the pectoralis major fascia, while also using this to define the lateral infra mammary fold. When the flap is inset vertically, Zone III occupies the lower part of the breast. This can be tucked/folded underneath and left partially de-epithelialized on the chest wall to improve breast projection [23].

#### 3.2.3. Breast Projection

**Figure 5:**
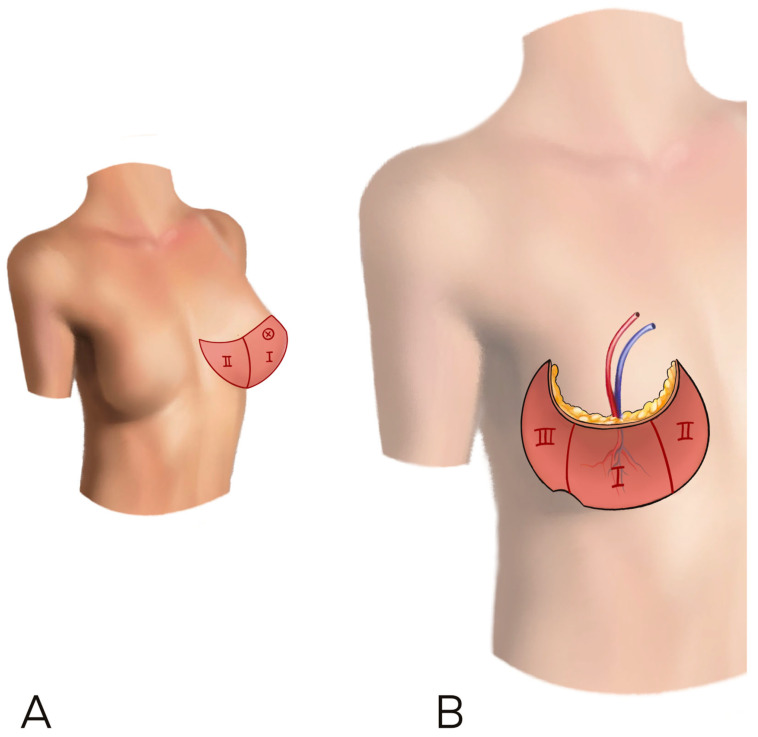
(**A**,**B**) In order to increase flap projection, a coning procedure enhances the antero-posterior dimension of the flap by reducing flap base. This can be obtained by suturing together the zone II and III of the flap.

A coning procedure enhances the antero-posterior dimension of the flap by reducing the flap base [8] (Figure 5). Projection can be achieved by suturing together zones II and III and maintaining zone I centrally within the site of maximum flap projection. Given that the umbilical part of the flap is usually the thickest, this is predominantly placed at the level of the IMF by keeping the flap positioned horizontally on the chest and rotating the flap 180°; zone III will therefore be medial when the flap is raised on a contralateral perforator and towards the axilla when the perforator is ipsilateral. When further projection of the lower pole is desirable, removing a wedge of skin around the umbilicus and suturing the remaining pillars together can improve the projection of the lower half of the flap [9]. When using a vertically inset flap to improve ptosis that has been rotated by 90° for inset, the thickest portion of the flap (around the umbilicus/Zone I) is set at the nipple region to improve maximum projection in this area. The umbilical part of the flap is often positioned medially, as suggested by Uda, but has also been described as positioned laterally, as suggested by Williams [15,22].

#### 3.2.4. Upper Pole Fullness

Upper pole fullness becomes critical in patients with a contralateral prosthetic reconstruction or augmented breast. A vertical inset can usually provide a reasonable upper pole volume and should be preferred over the horizontal technique, which contributes minimally to the upper pole and often requires fat transfer at a later stage [9].

Gravannis et al. described the dual plane insetting technique: the pectoralis major is split at the level of the mastectomy scar, and a submuscular pocket is created, elevating the upper mastectomy skin en bloc with the muscle. After revascularization, the flap is inset behind the pectoral in the upper part and in front of the pectoral in the lower part, thus creating a smooth transition at the upper pole and preventing stepping or depressions [21].

### 3.3. Stacked/Conjoined Flaps

Bipedicled DIEP flaps can be either stacked, when the abdominal flap is divided into two hemi-abdominal separate flaps, or conjoined, when the abdominal flap remains intact over two pedicles.

Stacked/conjoined flaps represent the ideal solution in a number of scenarios to improve aesthetic outcomes. Initially, stacked flaps were introduced when the presence of scars from previous surgery jeopardized the use of a single pedicle abdominal flap. Intraflap anastomosis or bipedicled perfusion was then necessary to avoid flap vascular insufficiency [36,37]. Combining both sides of the abdominal pannus has become progressively more popular in large-breasted, slim women when the estimated volume of a single DIEP flap is not considered sufficient to reconstruct the whole breast mound or in patients declining contralateral breast reduction [28]. Bipedicled flaps are also often ideal in delayed reconstruction when a greater amount of skin is needed to recreate the breast mound with sufficient ptosis while avoiding creating a “patch” appearance to the reconstruction by leaving native chest wall skin above the IMF. In such cases, bipedicled flaps may represent the best option to meet the reconstructive aims of recreating breast volume, projection, and natural ptosis [38].

When bipedicle flaps are left conjoined, they can either be folded or coned. The authors’ inset principles for bipedicle flaps resemble what was previously described for single flap inset and aim to achieve all the same desired reconstructive outcomes (ptosis, projection, and fullness). Salibian et al. described 36 conjoined DIEP flaps: they harvested the entire abdomen on two pedicles, which were then connected anterogradely and retrogradely to the internal mammary artery [29]. Flap coning can be achieved by suturing the cranial margin of the abdominal tissue to itself to form a cone [6]. It is ideal to improve breast projection, while breast width can be tailored with symmetrical or asymmetrical coning and varying degrees of flap rotation to match the native breast footprint. The ideal circumstances for using this technique are when the abdominal pannus is thick enough to permit coning without collapse of projection. In extremely thin patients, flap folding is usually more effective in achieving adequate projection [27].

The abdominal pannus can be folded in the midline (symmetrically) or off the midline (asymmetrically). Flap pedicles are kept inside the folded abdominal pannus. The vertical inset in this case is particularly useful to reconstruct a narrow breast, and the central folded part of the flap is used to improve lower pole projection [30].

Stacked flaps, being separate, can have a number of horizontal or vertical adjacent flap variations, further adding flexibility to match the contralateral breast size and shape. The potential anastomotic configurations of stacked flaps are multiple, with either one flap on top of another buried flap and intra-flap anastomoses or one flap on top of the other with both flaps vascularized using independent extra-flap anastomoses (e.g., IMA/V antegrade and retrograde).

Dellacroce et al. and Garrido used two stacked hemiabdominal flaps layered on top of each other to increase the breast projection [11,19]. A vertical inset was chosen, with the umbilical part of each flap directed towards the lower pole. When considering flap composition, the abdominal pannus can be divided at the midline (symmetrically) or off midline (asymmetrically): the larger flap can improve breast height and ptosis (vertical inset) or breast width (horizontal inset), while the smaller flap can improve breast projection as an augmentation flap [6]. To optimize freedom of inset and flap mobility, the more superficial flap is anastomosed to a run-off bransition at the upper pole and preventing step-pan intra-flap anastomose [26].

### 3.4. Patient Body Type

Razzano et al. described two main categories of the patient’s abdomen: the skin-predominant abdomen and the fat-predominant abdomen, based on the thickness of the subcutaneous tissue measured with a ruler in the central part of the flap [9,39]. When dealing with a fat-predominant abdomen, only a small part of the flap can be folded inferiorly or laterally; a horizontal inset is easier to perform and provides good definition of the lateral border with better fullness medially in the cleavage area [24].

Flaps from skin-predominant abdomens are more pliable and therefore more useful for reconstructing a ptotic breast when inset vertically with 90 degrees of rotation, especially those with grade 2 or 3 ptosis following weight loss or pregnancy. The degree of breast ptosis desired will influence the rotation of the flap (less ptosis in horizontal insets versus more ptosis in vertical orientation) [23]. The possibility to enhance ptosis and tailor the flap may influence the patient’s wishes regarding contralateral symmetrization, which is often proposed but may potentially be avoided if clear inset and shaping principles are followed.

When raising abdominal free flaps in thin patients with less skin laxity, harvesting a flap with sufficient vertical height can be difficult and can lead to hypertrophic scarring at the donor site, high-riding scars, and post-operative abdominal stiffness, which can be long-lasting. Uda et al. proposed a flap design modification (Sombrero shape) including a fan-shaped adipose flap just above the center of the cephalic portion of the flap, allowing the skin paddle to be narrower than a conventional flap [22]. As mentioned above, stacked/bipedicled flaps are another popular option in these cases.

### 3.5. Predesigned Insetting

While most of the papers described flap inset in a freehand fashion, a predesigned flap based on pre-operative imaging or on the contralateral breast could theoretically be effective and reduce the operative time.

Patel et al. described a paper template created by wrapping the opposite breast with an inverted V-shape triangular flap designed on the inferior mastectomy skin flap. With this technique, it is possible to reduce the vertical height of the abdominal flap, reduce the tension on the scar, and reduce the risk of donor site morbidity. The template can then be copied onto sterile paper during surgery and placed on the abdomen upright or upside-down, depending on which side has the most reliable pedicle. By using the template and retaining the segment of the inferior mastectomy skin flap, the authors were able to produce a reconstruction of sufficient volume with adequate tissue in both the upper and lower poles of the breast and a good conical breast shape [16].

A 3D imaging CT scan was used by Gelati et al. to recreate DIEP sizer models based on patient breast and thorax measurements: the healthy breast is scanned, the image obtained is mirrored on the contralateral side, and from this 3D image, the authors were able to create a personalized negative mold for use in the operating room to better shape the abdominal flap. Once the flap is raised, it is positioned inside the sizer, shaped to fit this mold, and then sutured into position [31].

Predesigning the flap directly on the abdomen has been proposed by Dyonyssion using the Boorman method: a template is prepared based on the dimensions of the contralateral breast [20]. A mirror image of the mastectomy scar is drawn on the normal breast. The skin envelope is estimated using the medial and lateral markings of the mastectomy scar line and the projection of the meridian line at the inframammary fold. The highest extent of the normal breast tissue above the mirrored mastectomy scar is calculated and will correspond to the buried part of the flap. The new breast cone is created by excision of a wedge on the pubic part of the abdominal flap, the limbs of which are equal to the distance of the contralateral nipple to the inframammary fold. All measurements are placed on a two-dimensional template that is transposed over the abdomen. The tissue outside of the template will be discarded.

### 3.6. Complications

According to the studies included in this review, fat necrosis was the most common complication (107 cases out of 1450, 7.3%). Revision of the microsurgical anastomosis was performed in 29 cases (2%). Partial flap loss was reported in 6 patients (0.4%), while complete flap loss occurred in 9 patients (0.7%). Complications are presented in Table 2.

## 4. Discussion

Nowadays, the ultimate goal of microsurgical breast reconstruction is not merely the effective transfer of vascularized tissue but the achievement of a natural, symmetric appearance. Autologous breast reconstructions using abdominal flaps are associated with high patient satisfaction when compared to prosthetic reconstructions [40].

The literature regarding free tissue transfer for breast reconstruction focuses mostly on safe tissue transfer data and complication rates, with flap design, shaping, and inset mostly neglected. The paucity of papers on flap design in the literature reflects the need for clear guidance on flap insetting procedures to match the width, fullness, ptosis, and projection of the contralateral breast.

Ideal flap positioning should follow body image and contralateral breast properties. Typically, in the aging breast, the lower pole tends to be more voluminous than the upper pole, while the lateral pole is typically more voluminous than the medial pole. In the reconstruction, the lower pole should ideally be full enough to achieve a natural shape, which is often prioritized in volume deployment. This leads to a neglected sub-clavicular area, which often results in a depression or stepping deformity requiring fat grafting. Interestingly, the lateral pole may not need to be particularly enhanced since the flap tends to slide down laterally over time [41].

For most patients who seek breast reconstruction, the contralateral breast is more likely to be teardrop-shaped and vertically elongated due to the effects of aging. Therefore, to mimic the natural breast, it is more intuitive to place the flap vertically (or slightly oblique in delayed reconstruction when mastectomy scars may be oblique and long). Indeed, when possible, a vertical inset often provides good volume to both the lower and upper poles, reducing steps or depressions [9]. The horizontal inset facilitates the provision of a good amount of tissue to the lower pole but contributes almost nothing to the upper pole and too much to the lateral pole, often necessitating liposuction and fat grafting at a later stage [23].

When planning fat grafting and liposuction secondary revisions, remodeling of the flap (such as lateral sling suspension or IMF position changing) is extremely useful to improve breast shape [42,43]. A careful initial assessment of the contralateral breast and body habitus is critical in achieving breast balance and harmony [44]. The principles of shaping (coning, folding, and stacking flaps) need to be considered to orient the flap correctly. Despite being the most suitable for a natural teardrop-like shape, the vertical flap setting has drawbacks. In secondary reconstruction, greater breast width and tissue replacement may be required [24]. This means that a purely vertically inset abdominal flap in a delayed setting will need to have a considerable cranio-caudal flap width to close the chest defect from medial to lateral. The vertical inset also requires the use of nearly the entirety of zones I, II, and III, especially if ptosis is desirable. A double-pedicled flap may then be a better option to optimize volume and shape without vascular perfusion issues, despite being technically more challenging.

This review suggests that the insetting technique in microsurgical breast reconstruction should be guided by three pillars: type of mastectomy/reconstruction timing, contralateral breast shape/size, and patient body type.

The type of mastectomy and the reconstruction timing will define the amount of skin required. When the skin envelope is maintained, a buried flap gives the best aesthetic result. In delayed procedures, a single aesthetic unit reconstruction provides better scarring.

The volume and shape of the contralateral breast should define the best flap orientation on the chest wall to match breast width, ptosis, projection, and upper pole fullness as closely as possible.

The patient’s body shape (i.e., slim, large-breasted women declining contralateral reductions) defines the need for conjoined/stacked flaps and influences flap folding and rotation decisions.

Interestingly, we could not find any flap insetting technique focusing on autologous reconstruction following previous implant-based reconstruction. In such patients, the presence of the implant capsule should be addressed, which may open up unique inset possibilities.

This review highlights that a number of authors are using templates based on the contralateral breast. These tools may further facilitate inset and shaping decisions.

The complications analysis performed on the included literature showed a lower incidence of complications compared to previous literature focusing on abdominal-based autologous breast reconstruction. This could be due to the fact that in most of the papers, the analysis of flap-related complications was not a primary outcome of the investigation, and therefore the real number of complications (e.g., microsurgical salvage procedures) may have been underestimated [45,46].

There is generally a paucity of literature describing a systematic approach to flap inset, which represents a limitation of this study. However, the principles described should help the reconstructive microsurgeon follow a rational pathway to ensure natural, aesthetic results.

## 5. Conclusions

Flap shaping and inset during breast reconstruction are fundamental steps in any reconstructive procedure. Despite the low evidence in the current literature, this systematic review provides a framework to guide the surgeon’s decision-making and optimize the aesthetic outcomes of abdominal-based free flap breast reconstruction.

## Figures and Tables

**Table 1:** Summary of studies on free flap inset for breast reconstruction (Listed by Year of Publication).

Author (First Listed), Year	Study Design	No. of Patients	Geographic Location	Age of Patients (y),	Delayed-Immed	Type of Flap	Flap Insetting	Recipient Vessels	Indications Proposed by the Authors	General Outcome Including Satisfaction
Blondeel et al., 1994 [5]	Case Report	1 patient, 2 stacked flaps	Belgium	37	1 delayed	bipedicled “stacked” DIEP		IMA	Midline abdominal scar in patient	good shape, size and projection
Garrido and Ramakrishnan 2002 [11]	Case Report	1 patient, 2 stacked flaps	UK	69	1 immediate	Stacked hemi-DIEPs	Second flap on top of the first totally buried flap. Fixation with absorbable sutures.	TDA and intraflap	Midline abdominal scar in patients with a D cup or more	Excellent cosmetic outcome and final volume. Microsurgical complexity. Serial anastomoses curb the necessity of monitoring the buried flap
Pülzl et al., 2005 [12]	Retrospective study	12 patients, 12 flaps	Austria	51	12 delayed	MS-TRAM 6 DIEP 1 SIEA flaps	Deepithelialized tissue from scar to IMF. Flap instead of the IMF. Ptosis adjusted by insetting a lower or greater amount of flap skin. Rest of flap buried under the mastectomy flap	IMA	All secondary reconstructions, including contralateral moderate ptosis	10 good, 2 fair aesthetic outcomes (evaluation by 2 independent surgeons)
Liao et al., 2005 [13]	Case Report	1 patient, 1 flap	Taiwan	52	1 delayed	DIEP flap	Flap turned 180 degrees, with the cranial part facing the new IMF. Cranial part of the flap is undermined. Skin paddle relocated superiorly to shift the NAC inferiorly.	IMA	Poland Syndrome, types B and D (chest depression)	Satisfactory aspect recreating lat axillary line and IMF
Cheng MH et al., 2006 [14]	Retrospective study	73 patients, 74 flaps	Taiwan	44.2	25 immediate, 49 delayed	DIEP (with zone IV)	Downward inset with croissant shape: the cephalic border of the flap points downward, pointing to the inframammary fold, with the umbilical located on the lateral end of the IMF. Zone IV (never discharged except for one case) located in the superior-lateral area	IMA 69; TDA 5	Thin patients or patients with controlateral breast pendulus and large breasts (with no desire of breast reduction)	Inclusion of zone IV is highly reliable and provides superior aesthetic outcomes and high satisfaction
Williams et al., 2008 [15]	Retrospective study	10	USA	53.7 years (range, 44.1–61.4)	10 immediate 2 delayed	free TRAM with immediate nipple reconstruction	Inset with a wrapping flap into the cone, umbilical downwards/lateral, zone III (according to Harp) in the axilla. DIEP inset oblique center of cone (most projected part) will be used for nipple recon bilobed with fishtail flap	IMA	Spares an additional surgery for nipple recon (minimizing the number of procedures,)	No difference in subjective cosmetic ratings between delayed and immediate NAC reconstruction
Patel et al., 2008 [16]	Retrospective study	21	UK	N/A	Delayed	Free TRAM	Pre-design the flap using a template created from the opposite breast. Triangular caudally-based mastectomy flap, the cranial skin of the DIEP is sutured to the triangle	IMA	Large ptotic breasts, natura Minimize abdominal wound morbidity (less vertical height)	without the need to raise a very large abdominal flap
Scholz T et al., 2008 [17]	Retrospective study	72 patients, 106 flaps	USA	51.5	Immediate	Free TRAM (94), DIEP (12)	Insetting the flap into a vertical skin pattern of skin sparing-mastectomy. All flap is di-epithelized except for a monitor on the neo-areola	IMA	Stage 0, I, II of AJCC Cancer Staging	Improvement of the aesthetic outcome without compromising oncologic safety Elimination of the disharmony between skin flap and breast volume in the vertical direction while respecting the inframammary crease. Produces a youthful, symmetrical conical breast shape with medial fullness
Bozikov K et al., 2009 [18]	Retrospective study, Single surgeon	100 patients, 100 flaps	Slovenia	NA	57 Immediate, 43 Delayed	DIEP based on contralateral deep inferior epigastric vessels (from 1 to 4 perforators)	Horizontal straight inset: the umbilical border of the flap was positioned at the cranial part of the chest defect, zone III being inset medially and zone II laterally. Entire zone IV discarded (Hartrampf’s classification)	IMA	Harvesting flap on multiple perforators reduces flap fat necrosis. BMI> or = 30 is a risk factor for fat necrosis. Flap zone III fat necrosis related to harvesting on a single lateral row perforator (and vice versa, zone II-medial row)	94% flap success
DellaCroce et al., 2010 [19]	Retrospective study	55 patients, 110 flaps	USA	48	27 immediate 19 delayed	55 bipedicled stacked DIEPs	Stacked flap in series. Flap 1 connects to IMAV, and flap 2 is connected to flap 1 by intra-flap “chain” anastomosis through a branch. Primary flap is kept outside, and flap 2 is buried. Flaps are generally separated, especially in delayed, to avoid a squared-off lower pole	IMA	Patients where single hemi-abdomen is bw 1/3 and ½ of desired final breast volume, need for zone IV	Superlative aesthetic outcomes with high patient satisfaction
Dionyssiou et al., 2014 [20]	Case Report	1	UK	52	1 delayed	DIEP flap	Pre- or post-operative mirroring of the opposite breast template performed, and the template is transposed over the abdomen and centralized around the selected perforators according to CT scan + angiography	IMA	(1) operative time spent for shaping and insetting is significantly reduced, (2) early excision of the unnecessary parts of the abdominal flap avoids the intraoperative and postoperative flap congestion , (3) decreasing the overall operative time reduces complications , and (4) the immediately produced breast symmetry significantly reduces the need for secondary symmetrization operations.	Group A: very good and good = 53.3% Group B: very good and good = 88.8%
Gravanis et al., 2015 [21]	Retrospective study	50 (Group a = 25; Group b = 25)	Greece	Group A: 41.1 +/− 1.5 B: 41.8 +/− 1.1	Delayed	DIEP	(a) Single Plane: Only upper pole of DIEP is de-epitheliazed. Insetting above the muscle, and the lower part of mastectomy skin is de-epeithelized. Three key sutures (upper-lateral, midclavicular, and lower-medial) Dual Plane (b) (upper part of flap is de-epitheized under the pec muscle)	IMA	Delayed Unilateral reconstruction Prevents ptosis Increases upper pole fullness	Dual plane group achieved a significantly higher score for a non-disruptive superior scar and non-disruptive superior mastectomy skin (Less scar/diep demarcation line) The outline of the breast is smooth and natural in appearance. Overall breast appearance,” the dual plane reconstruction group scored 86 +/− 1.5, and the single plane reconstructions scored 72.2 +/− 1.9. This difference was extremely statistically significant (*p* < 0.0001).
Uda et al., 2016 [22]	Retrospective study	62 patients	Japan	49	Immediate (only Skin sparing mastectomy)	35 DIEP and 27 bipedicled DIEP	Flap placed 90 degrees medially and mounted vertically. The thickest portion of the flap around the umbilicus is set at the nipple and areolar region, and the ipsilateral lateral portion of the flap fill the defect of décolleté and axillary tail. The contralateral portion of the flap is folded inward to create lower pole fullness. If double-pedicled flaps: intraflap crossover anastomosis before anterograde end-to-end anastomosis OR anterograde and retrograde double end-to-end anastomosis.	IMA	Slender Asian patients: vertical flap setting with S-flap (medial fan-shaped adipose flap) West patients: C-flap	the vertical flap setting imposes the use of nearly the entirety of zone II and sometimes zone IV and the flap must often be elevated as a double-pedicled flap Good satisfaction comparative to conventional flap only for morbidity donor site
Patel et al., 2016 [6]	Retrospective study	25 patients	USA	48	Immediate	Bipedicled (14) and Stacked (11) DIEP	Folded: flap left undivided but folded at the midline (symmetrically) or off midline (asymmetrically) Divided: abdominal pannus is divided and skin paddle layered on top of each other Coned: cranial margin of abdominal tissue sutured togheter Divided and folded: abdominal pannus divided and each flap folded	TDA 67%, IMA 32%,	Folded: tall narrow breast, more volume in the inferior pole Divided: if set horizontally -> increased breast base Coned: projected breast with large inferior pole Divided and coned: moderate projection	Stacked and bipedicled flaps are more demanding but less fat necrosis is seen
Jeong et al., 2016 [23]	Retrospective study	274 patients	South Korea	45	250 immediate, 24 delayed	DIEP	Horizontal inset: umbilical site flap directed downwards, zone II medial Vertical inset: umbilical side lateral, zone II lowest part of the breast	IMA and TDA	Horizontal inset: more volume at the lower pole Vertical inset: balanced volume distribution,	Higher symmetry in vertical inset, lateral excess in horizontal inset with volume deficiency in upper pole
Gravannis et al., 2016 [24]	Prospective study, single surgeon	42 patients	Greece	42	Delayed, RT	DIEP	Dual plane: the flap in inset over the di-epithelized mastectomy lower flap and at the upper pole under a myocutaneous flap including the pec maj. PDS sutures (parasternal, midline and ant axil line) between flap scarpa fascia and undersurface of pec major	IMA	Replacement of poor quality mastectomy skin, optimal IMF position, reduced skin problems at the upper mastectomy flap	Fullness upper pole and minimal ptosis overtime. Improved social and sexual life, high satisfaction for aesthetic result
De La Parra Marquez. 2018 [25]	Retrospective study	8	Mexico	Mean 45 years old, (42–50 years; SD = 3.30)	Delayed (after failed immediate implant) (Immediate tertiary DIEP)	Deepithelialized DIEP	Deepithelialized flap is placed in the same pocket where the previous implant was; no new pocket or change of plane	IMA	Salvage for failed implant based reconstruction	Results in a soft and natural final breast shape.
Yu et al., 2020 [26]	Case Report	1 patient, 2 flaps	UK	47	1 delayed (tertiary)	DIEP + SIEA stacked	Both flaps vertically oriented (lat edge cranial) Controlat DIEP outside (antegrade micro on IMA/V), buried ipsilateral SIEA with dermis facing down to allow reaching IMA/V (retrograde)	IMA (anterograde and retrograde)		Pleasant result due to ideal flap orientation (both flaps with lateral part cranial and bulgy part in the lower pole)
Chang et al., 2016 [27]	Retrospective study	57 pt, 114 flaps	USA	49	21 immediate, 36 delayed	Dual pedicle flap (TRAM 42, DIEP 59, SIEA 12)	Lateral ends of the flap folded onto themselves, no rotation, horizontal	IMA antegrade and retrograde removing the rib, TDA (8)	Dual pedicle flap increases confort with free tissue transfer and high success rate	Valuable option when more volume is needed
Suh et al., 2019 [28]	Case report	1	South Korea	48	immediate	DIEP + SIEA contralat stacked	elliptical-shaped flap was inset with a 90° counterclockwise rotation, and the lower one-third of the flap was folded to create a projection	DIEA to IMA, SIEA to TDA	Large and ptotic breast in thin patient	Avoid bilateral abdominal fascia incision. SIEA short pedicle. LD flap can still be performed as salvage option.
Razzano et al., 2019 [9]	Prospective study	70	UK	55	70 immediate	DIEP (5 bipedicled)	Depending on contralat breast: If ptotic: vertical inset folding the inferior portion of the flap with 90° rotation If projected: horizontal inset, 0° rotation if ipsilateral DIEP, 180° rotation if contralateral, with lateral and inferior folding Fat abdomen: horizontal inset Slim abdomen: vertical inset	IMA and intraflap anastomosis	Flap inset changes depending on the controlat breast, perf position and type of abdomen	180-degree rotation provides the best possible projection, with the position of the umbilical vertical scar placed inferiorly 90-degree flap counterclockwise rotation allows the pedicle to be placed in a more medial position. When the breast base is large or projection and fullness of the upper pole are needed, rotate the flap 0 or 180 degrees, depending on the position of the perforators. When 90 degrees of rotation is chosen, better ptosis could be achieved by deepithelializing and folding the inferior marg
Salibian et al., 2020 [29]	Retrospective study	182 patients	USA	52	105 immediate, 77 delayed	DIEP stacked/conjoined (36), non-stacked (146)	In stacked: hemiflaps in the inferior and superior breast pole In conjoined: coning the flap while maintaining the base, flap rotation based on native footprint	IMA	Conjoined flap in lower BMI and prior irradiation	Patient with stacked flaps lower contralaterl simmetrisation
Pompei et al., 2020 [30]	Review + retrospective	28 flaps	UK	50	Immediate	Stacked DIEP flap	Calzone technique: flap folded in two in the back table on the horizontal axis, posterior side de-epitelized, other side partially or totally de-epit depending on skin envelop. The folded part stays inferior.	IMA antegrade and retrograde	Flap augmentation technique, enhancing lower pole projection	
Gelati et al., 2020 [31]	Retrospective study	24 pt with DIEP sizers vs. 24	Italy	50	21 Delayed, 3 immediate	DIEP	Virtual model using Geomagic Xsoftware to construct the DIEP sizers. the abdominal flap is located inside the DIEP sizer. The flap is fixed onto the thorax skin, the anastomoses are made, and afterwards, proceed to modeling by trimming the flap in the DIEP sizer	IMA	10 different DIEP sizers were created based on the anthropometric measures of 15 patients who previously underwent DIEP reconstruction. Ideally, the contralat breast can be used as a 3D model to build the sizer.	Significative OP time difference between groups (faster with sizers).

Abb.: DIEP, deep inferior epigastric perforator; IMA, internal mammary artery; TRAM, transverse rectus abdominis muscle; MS-TRAM: muscle sparing transverse rectus abdominis muscle; SIEA, superficial inferior epigastric artery; TDA, toraco-dorsal artery; BMI, body mass index; IMF, inframammary fold; RT, radiotherapy; NAC, nipple areola complex; NA, not applicable.

**Table 2:** Summary of Complications.

Author	Number of Patients	Number of Flaps	Type	Complication	Complication
				Breast	Donor Site
Blondeel et al., 1994 [5]	1	2	Bipediceld stacked DIEP	0	-
Garrido and Ramakrishnan 2002 [11]	1	2	Stacked hemi-DIEPs	1 venous congestion solved	-
Pülzl et al., 2005 [12]	12	12	5 MS-TRAM,6 DIEP, 1 SIEA	1 hematoma	-
Liao et al., 2005 [13]	1	1	DIEP flap	0	-
Cheng MH et al., 2006 [14]	73	74	DIEP flap	2 partial flap loss	-
				10 Liponecrosis	
				1 venous congestion solved	
Williams et al., 2008 [15]	10	12	Free TRAM	0	-
Patel et al., 2008 [16]	21	21	Free TRAM	0	3 cases of minor abdominal wound infection
Santanelli et al., 2008	4	4	Free vertical DIEP flap	0	-
Scholz T et al., 2008 [17]	72	106 (34 BILATERAL)	Free TRAM (94), DIEP (12)	4 fat necrosis	3 Fat necrosis
				1 flap site infection	2 Wound dehiscence
				1 venous occlusion requiring revision	3 Wound infection
					1 Seroma
Bozikov K et al., 2009 [18]	100	100	DIEP	6 total flap Loss	-
				37 liponecrosis	
				20 anastomosis revision	
DellaCroce et al., 2010 [19]	55	110	55 bipedicled stacked DIEPs	3 hematoma including 1 requiring surgical evacuation	-
Dionyssiou et al., 2014 [20]	32	32	MS-TRAM(7) DIEP(25)	3 venous thrombosis (all in DIEP)	-
Gravanis et al., 2015 [21]	50	50	DIEP	none	-
Uda et al., 2016 [22]	62	89	35 DIEP and 27 bipedicled DIEP	2 total flap loss	2 abdominal wound healing
				3 partial flap loss	14 abdominal seroma
				8 partial fat necrosis	5 bulging
				3 hematomas	
				3 infections	
				2 mastectomy flap necrosis	
Patel et al., 2016 [6]	25	50	Bipedicled (14) and Stacked (11) DIEP	1 Flap loss	-
				2 Hematoma	
Jeong et al., 2016 [23]	274	274	DIEP and MS-TRAM	NR	-
Gravannis et al., 2016 [24]	42	42	DIEP	0	-
De La Parra Marquez. 2018 [25]	8	10	DIEP	3 seroma	-
Yu et al., 2020 [26]	1	2	DIEP + SIEA stacked	0	-
Chang et al., 2016 [27]	57	103	Bipedicled flap (TRAM, DIEP, SIEA)	4 Fat necrosis (all pedicled TRAM)	2 seroma
		(46 dual)		1 Seroma	2 Abdominal bulge (from pedicled TRAM)
		11 p-TRAM + freeflap		1 Hematoma	1 infection
				6 Wound healing	2 wound healing
				1 Partial flap loss (dual pedicle)	
				3 Infection	
Suh et al., 2019 [28]	1	2	DIEP + SIEA contralat	0	-
Razzano et al., 2019 [9]	70	75	DIEP (5 bipedicled)	4 Fat necrosis	-
				14 Revision surgery	
Salibian et al., 2020 [29]	182	218	DIEP and MS-TRAM stacked/conjoined (36), non-stacked (146)	3 Fat necrosis (8%) in conjoined	-
				37 fat necrosis (25%) in non-conjoined	
Pompei et al., 2020 [30]	28	56	Bipedicled DIEP	0 flap necrosis	-
				4 fat necrosis	
Gelati et al., 2020 [31]	48	59	DIEP	0	-

Abbreviations: DIEP, deep inferior epigastric perforator; TRAM, transverse rectus abdominis muscle; MS-TRAM: muscle sparing transverse rectus abdominis muscle; SIEA, superficial inferior epigastric artery.

## Data Availability

No new data were created or analyzed in this study. Data sharing is not applicable to this article.
